# Criminality in patients with autoimmune encephalitis: A case series

**DOI:** 10.1111/ene.16197

**Published:** 2024-01-08

**Authors:** Sophia Michael, James Varley, Robyn Williams, Tomasz Bajorek, Ava Easton, Sarosh R. Irani

**Affiliations:** ^1^ Oxford Autoimmune Neurology Group, Nuffield Department of Clinical Neurosciences University of Oxford Oxford UK; ^2^ Department of Neurology, John Radcliffe Hospital Oxford University Hospitals Oxford UK; ^3^ Departments of Neurology and Neurosciences Mayo Clinic Jacksonville Florida USA; ^4^ Encephalitis Society Malton UK; ^5^ Department of Clinical Infection, Microbiology, and Immunology University of Liverpool Liverpool UK

**Keywords:** antibody, behaviour, crimes, encephalitis, limbic

## Abstract

**Background and purpose:**

Despite it being an immunotherapy‐responsive neurological syndrome, patients with autoimmune encephalitis (AE) frequently exhibit residual neurobehavioural features. Here, we report criminal behaviours as a serious and novel postencephalitic association.

**Methods:**

This retrospective cohort study included 301 AE patients. Five of who committed crimes underwent direct assessments and records review alongside autoantibody studies.

**Results:**

Five of 301 patients (1.7%) with AE exhibited criminal behaviours, which included viewing child pornography (*n* = 3), repeated shoplifting, and conspiracy to commit murder. All five were adult males, with LGI1 autoantibodies (*n* = 3), CASPR2 autoantibodies, or seronegative AE. None had evidence of premorbid antisocial personality traits or psychiatric disorders. Criminal behaviours began a median of 18 months (range = 15 months–12 years) after encephalitis onset. At the time of crimes, two patients were immunotherapy‐naïve, three had been administered late immunotherapies (at 5 weeks–4 months), many neurobehavioural features persisted, and new obsessive behaviours had appeared. However, cognition, seizure, and disability measures had improved, alongside reduced autoantibody levels.

**Conclusions:**

Criminal behaviours are a rare, novel, and stigmatizing residual neurobehavioural phenotype in AE, with significant social and legal implications. With caution towards overattribution, we suggest they occur as part of a postencephalitis limbic neurobehavioural syndrome.

## INTRODUCTION

Patients with autoimmune encephalitis (AE) often present with seizures, amnesia, and various additional neurobehavioural deficits including psychosis, affective disturbances, and emotionality [1]. Many forms of AE are autoantibody‐mediated. The commonest autoantibodies target leucine‐rich glioma‐inactivated 1 (LGI1) and the N‐methyl D‐aspartate receptor (NMDAR). Other patients show a similar clinical phenotype without a known antigenic target, termed "seronegative." Despite showing objective improvements after immunotherapies [2, 3, 4], almost all AE patients report residual neuropsychiatric features affecting sleep, depression, anxiety, and fatigue, markedly impairing their quality of life (QoL) [5, 6].

Here, we describe clinical features, focused on residual behavioural features, from a small but important group of AE patients who committed crimes. By reporting timing of administered immunotherapies, clinical features over time, and the marked social consequences, alongside autoantibody levels, we cautiously propose criminality may be a rare, residual, and markedly impactful feature of AE that relates to an established disturbance of limbic neurocircuitry without evidence of an immunological relapse.

## METHODS

A total of 301 patients were seen in the Oxford Autoimmune Neurology Clinic, UK, as clinical or research referrals between 2014 and 2021, with antibodies against LGI1 (*n* = 121), the NMDAR (*n* = 54), or contactin‐associated protein 2‐like (CASPR2; *n* = 46), other rarer targets or seronegative AE (*n* = 25), or non‐autoimmune diagnoses (*n* = 55). From five with criminal behaviours, detailed clinical phenotyping was performed using direct patient, partner, and carer interviews, case note review, and measures of cognition (Addenbrooke Cognitive Examination [ACE]), mood (Hospital Anxiety and Depression Scale [HADS]), and disability (modified Rankin Scale [mRS]). Mann–Whitney *U*‐tests were used for statistics. All patients provided written informed consent (Research Ethics Committee approval 16/YH/0013).

## RESULTS

Five of 301 (1.7%) patients reported criminal behaviours, which included viewing online child pornography (*n* = 3, in one case also indecent public exposure), repeated shoplifting (*n* = 1), and conspiracy to commit murder (*n* = 1). The three patients viewing illegal materials were formally prosecuted, and the theft led to a ban from the relevant supermarket.

**FIGURE 1:**
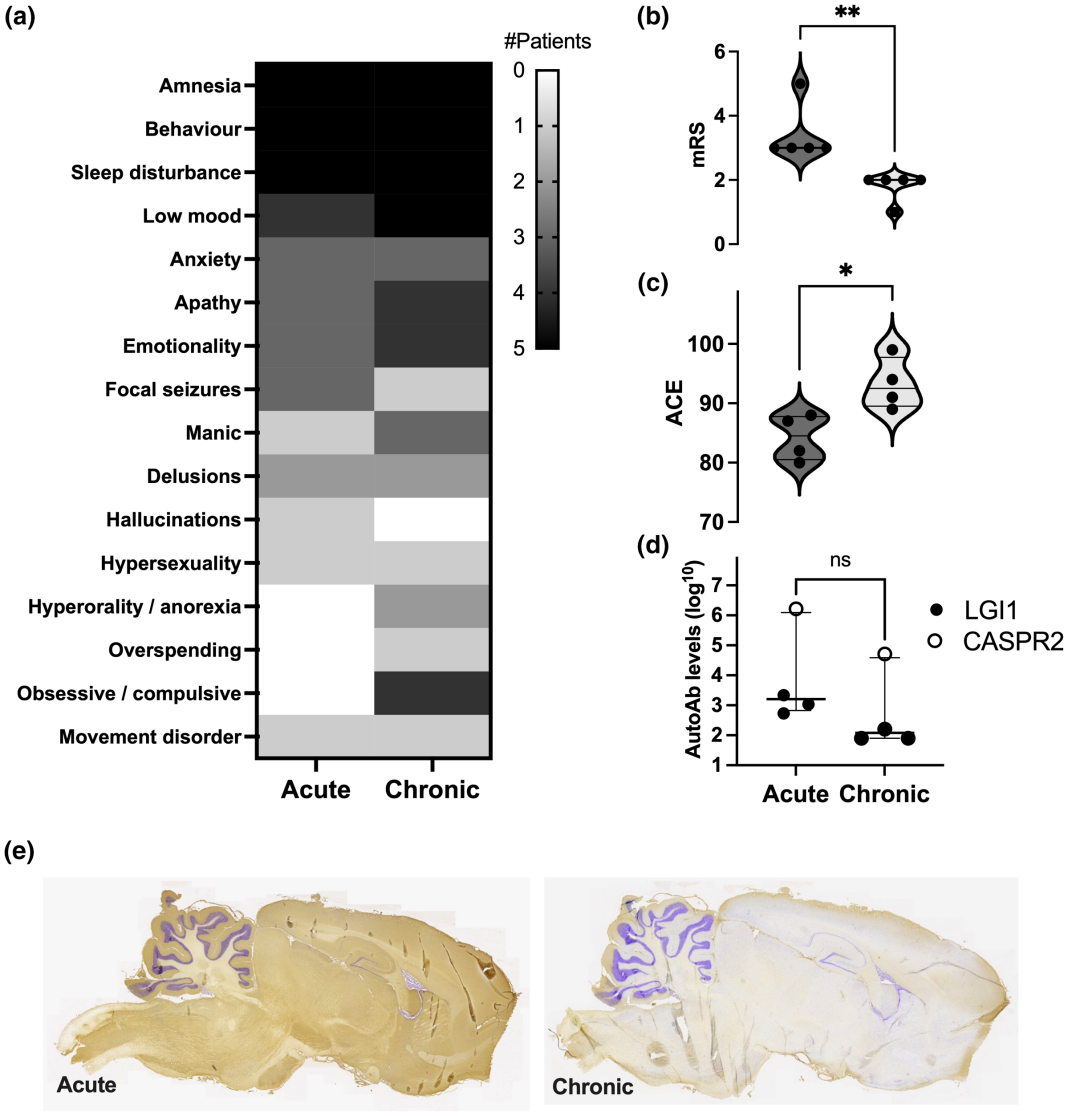
Acute and chronic features associated with five patients with autoimmune encephalitis (AE) who committed crimes. (a–d) Number of patients (0–5) with each of 16 features (a) and their modified Rankin Scale (mRS) scores (b; **p = 0.0079, Mann–Whitney test), Addenbrooke Cognitive Examination (ACE) results (c; *p = 0.03, Mann–Whitney test), and autoantibody levels (d; measured by live cell‐based assay end point dilutions; shown as a log scale) in both acute and chronic phases of AE. (e) Representative examples of acute and chronic serum IgG binding to rodent brain sections (from Patient #1). AutoAb, autoantibody; ns, not significant.

Their diagnoses were LGI1‐antibody encephalitis (*n* = 3), CASPR2‐antibody encephalitis (*n* = 1), and seronegative AE (*n* = 1). All were male, with onset of AE in their 40s–60s (Table 1). Acute features were typical for AE (Figure 1a): all with amnesia, behavioural disturbances, especially irritability and aggression, and insomnia; four showed affective features (low mood and anxiety); three exhibited apathy, emotionality, or seizures, and two displayed delusions and/or mania. Visual hallucinations or hypersexuality were only reported in one patient each. None showed obsessive–compulsive tendencies acutely, and none had premorbid antisocial behaviours, criminal convictions, or psychiatric disorders.

**TABLE 1:** Clinical characteristics of five autoimmune encephalitis patients who committed crimes.

Case	1	2	3	4	5
Age at onset	60s	40s	50s	50s	60s
Gender	Male	Male	Male	Male	Male
Autoantibody	CASPR2	Seronegative	LGI1	LGI1	LGI1
Criminal/near criminal offence	Viewing child pornography; indecent public exposure	Viewing child pornography	Viewing child pornography	Repeated shoplifting	Plotted to kill wife
Brain imaging	Normal CT	High signal in corpus callosum splenium	Normal MRI	Bitemporal T2 hyperintensities	Normal MRI
CSF findings	NA	59 white cells; protein (1.2 g/L)	Normal	NA	Normal
EEG findings	NA	Frontotemporal slow waves	Normal	NA	Diffuse slowing
Tumour	Yes: likely SCLC^a^	No	No	No	No
Time to immunotherapy	13 years	5 weeks	4 months	4 months	25 months
Time to criminal behaviour	12 years	15 months	6 years	18 months	18 months
Time to last follow‐up	13 years	5 years	7 years	5 years	7 years
Immunotherapies upon diagnosis	Oral prednisolone	Intravenous methylprednisolone; oral prednisolone	Oral prednisolone; IVIG	Oral prednisolone; IVIG	Oral prednisolone; IVIG
Immunotherapies at time of crime	Never	Nil (stopped 9 months prior)	Nil (stopped 5 years prior)	Nil (stopped 1 year prior)	Never
Neurotropics at time of crime	Tetrabenazine, zopiclone, olanzapine	Nil	Sertraline	Nil	Nil

*Note*: Ages are intentionally approximated to preserve anonymity.

Abbreviations: CASPR2, contactin‐associated protein 2‐like; CSF, cerebrospinal fluid; CT, computed tomography; EEG, electroencephalographic; IVIG, intravenous immunoglobulins; LGI1, leucine‐rich glioma‐inactivated 1; MRI, magnetic resonance imaging; NA, not available; SCLC, small cell lung cancer.

^a^ On imaging, a lung tumour was diagnosed, although no biopsy was performed.

Criminal behaviours occurred at a median of 18 months (range = 15 months–12 years) after symptom onset. Two patients had not received immunotherapies before crimes were committed. The remaining three had lags from symptom onset to immunotherapy administration of 5 weeks (*n* = 1) or 4 months (*n* = 2). At the time of criminal activities, no patient was on immunotherapies, and two were receiving neurotropic drugs (Table 1).

In two patients, the criminal behaviours occurred over several weeks alongside development of multiple psychiatric features, including grandiose delusions, euphoria, irritability, and obsessive–compulsive behaviours. The latter included the repeated use of a mobile phone with frequent screen tapping, obsessions over car parking orientations, and compulsive shoplifting. In the other three patients, no new behavioural features emerged around the times of crimes. In all patients, no changes in seizures or cognition were observed around the periods of criminal activity, and reinvestigation with magnetic resonance imaging and cerebrospinal fluid (CSF) studies in four patients was unremarkable.

At latest follow‐up (median time = 7 years, range = 5–13 years), all five patients exhibited low mood (median HADS = 14, range = 7–32), insomnia, and behavioural disturbances, in particular agitation, irritability, and aggression. In four, obsessive–compulsive features, apathy, and emotionality were reported; three noted symptoms of anxiety; two presented with mania, hallucinations, and/or delusions and one with hypersexuality. One patient had persistent seizures and another a movement disorder. Despite these features, all showed good functional outcomes, with mRS ≤ 2 [2, 3] (improving from 3–5, *p* = 0.0079; Figure 1b), with ACE scores ≥ 89/100 (median = 93, range = 89–99), which had improved from disease nadir (*p* = 0.03; Figure 1c). One patient reoffended. None were treated with further immunotherapies or neurotropic drugs.

Upon confessing the crimes, all felt marked guilt and three of five patients were rejected by their families, necessitating new accommodation and social networks. One was later reintegrated into their original family although with ongoing domestic consequences.

At last follow‐up, LGI1‐ and CASPR2‐autoantibody levels had fallen approximately 30‐fold (Figure 1d) from first samples, with associated reductions in immunoglobulin G binding to limbic rodent brain regions (Figure 1e).

## DISCUSSION

We identify criminality in ~2% of a substantial AE cohort, occurring during the chronic phase in males in their fourth to sixth decades. Around the time of offences, these five subjects showed obsessive–compulsive behaviours, absent at disease onset, and, in two, new onset disinhibitory psychopathology. In addition, all patients showed persistent deficits in several domains of behaviour, memory, sleep, emotionality, and apathy. This was despite considerable and sustained improvements in traditional disability, seizure, and cognitive outcomes, with substantial reductions in autoantibody levels. None were offered medications, and only one reoffended. Hence, criminality occurred in AE patients with multiple residual neurobehavioural deficits without clinical or serological evidence of an active relapse, consistent with a postencephalitic phenomenon.

Criminality occurred in two patients (one with LGI1 and one with CASPR2 antibodies) who were immunotherapy‐naïve, and in three administered late immunotherapies (after 4 months in two). Such lags are increasingly unusual in clinical practice and exceed the ~30‐day threshold that predisposes to poor shorter term outcomes [2, 3, 4]. Therefore, we propose late and limited immunotherapies may be partly responsible for multiple residual neurocognitive syndromes, now including criminality. Given that two patients showed hypomania and disinhibition around the time of their criminality, it may be that vigilance for worsening neurobehavioural features can help identify future high‐risk patients and mitigate crimes.

None of our patients showed premorbid antisocial behaviours or criminal convictions, and they took minimal medications when crimes were committed. Hence, no obvious confounding factors were identified in ascribing criminality to AE. Yet, we continue to urge significant caution in this association, as accessing online pornography is common in elderly men, can frequently include violent or unlawful material [7, 8], and, overall, crimes are undertaken by ~0.2% of males older than 50 years [9, 10]. Hence, given our baseline demographic, this may represent a chance association, unrelated to AE.

Perhaps more likely, static postencephalitic lesions underlie the residual neurobehavioural features that manifested with criminality. This limbic–centric neurocircuitry is consistent with density of antigenic targets in the medial temporal lobe, and pattern of IgG reactivities (Figure 1e). Indeed, these regions are implicated in disinhibited behaviours, mania, and criminality in Parkinson disease, frontotemporal lobe dementias, and other neurological conditions [11, 12]. Hence, there is broad anatomical plausibility for these clinical observations.

As criminality led to formal prosecutions and major domestic, social, and legal consequences, this feature induced a markedly reduced QoL. Yet, mRS suggests "good" outcomes, indicating a need for more patient‐focused measures in AE [5, 6]. Furthermore, a joined‐up approach to multidisciplinary neurology and psychiatric care, alongside advice from societies experienced in AE, will greatly benefit these patients.

Study limitations include the referral bias of more immunotherapy‐resistant patients, the limited data available from medical assessments immediately at the time of crimes when legal proceedings took precedence, the lack of formal access to Police National Computer records, and the absence of sequential serum/CSF samples to exclude small rises in autoantibody levels.

In summary, criminality may represent a rare but life‐changing feature of AE whose occurrence may be modified with early immunotherapy administration. Although the serious and disturbing nature of crimes necessitate reluctance in definitively adding this to the range of postencephalitis syndrome features, timing, symptoms, and neuroanatomical considerations make this a plausible hypothesis that should be validated in future series.

## AUTHOR CONTRIBUTIONS

**Sarosh R. Irani:** Conceptualization; data curation; writing – original draft; visualization; formal analysis; writing – review and editing. **Sophia Michael:** Data curation; writing – review and editing; conceptualization; formal analysis. **James Varley:** Data curation; writing – review and editing. **Robyn Williams:** Data curation; formal analysis; writing – review and editing. **Tomasz Bajorek:** Conceptualization; data curation; writing – review and editing. **Ava Easton:** Conceptualization; data curation; writing – review and editing.

## FUNDING INFORMATION

This research was funded in whole or in part by a senior clinical fellowship from the Medical Research Council (MR/V007173/1), Wellcome Trust Fellowship (104079/Z/14/Z), BMA research grants Vera Down Grant (2013) and Margaret Temple Grant (2017), Epilepsy Research UK (P1201), UK‐US Fulbright Commission (MS Society research award), and National Institute for Health Research (NIHR) Oxford Biomedical Research Centre. For the purpose of open access, the author has applied a CC BY public copyright licence to any author accepted manuscript version arising from this submission. The views expressed are those of the author(s) and not necessarily those of the National Health Service, the NIHR, or the Department of Health.

## CONFLICT OF INTEREST STATEMENT

S.R.I. has received honoraria/research support from UCB, Immunovant, MedImmun, Roche, Janssen, Cerebral Therapeutics, ADC Therapeutics, Brain, CSL Behring, and ONO Pharma; receives licensed royalties on patent application WO/2010/046716 entitled "Neurological Autoimmune Disorders"; and has filed two other patents entitled “Diagnostic Method and Therapy” (WO2019211633 and US‐2021‐0071249‐A1; PCT application WO202189788A1) and “Biomarkers” (PCT/GB2022/050614 and WO202189788A1).

## Data Availability

The data that support the findings of this study are available on request from the corresponding author. The data are not publicly available due to privacy or ethical restrictions.
